# Fulminant Myocarditis in a Child Requiring Extracorporeal Cardiopulmonary Resuscitation: A Case Report

**DOI:** 10.7759/cureus.31561

**Published:** 2022-11-16

**Authors:** Takehara Sayuri, Ichibayashi Ryo, Watanabe Masayuki, Takatsuki Shinichi, Honda Mitsuru

**Affiliations:** 1 Critical Care Center, Toho University Medical Center, Tokyo, JPN; 2 Pediatrics, Toho University Medical Center, Tokyo, JPN

**Keywords:** extracorporeal cardiopulmonary resuscitation, out of hospital cardiac arrest, extracorporeal membrane oxygenation support, out-of-hospital cardiac arrest, fulminant myocarditis

## Abstract

Extracorporeal cardiopulmonary resuscitation (ECPR) has been rarely performed in children with out-of-hospital cardiac arrest (OHCA), and the evidence for the use of ECPR in OHCA is inadequate. Hence, the accumulation of data from each case of pediatric OHCA is important for establishing evidence. The patient was a 10-year-old, innately healthy girl. In the early morning on the day of admission, she had a sudden cardiac arrest and was rushed to the hospital by ambulance. On arrival at the hospital, the waveform on the electrocardiographic monitor indicated ventricular tachycardia (VT). Defibrillation was performed. But as the VT persisted, the emergency physician performed ECPR in the emergency department (ED). We diagnosed her with fulminant myocarditis by the preceding symptoms of common cold, rapid circulatory failure, echocardiographic findings, electrocardiographic changes, and hematologic test results showing elevated levels of myocardial component proteins. The patient was discharged without neurological sequelae on hospital day 25. We summarize the clinical characteristics based on an analysis of the clinical course.

## Introduction

According to the 2020 Japan Resuscitation Council (JRC) Guidelines, initiation of ECPR in children is considered for in-hospital cardiogenic cardiac arrest occurring in the intensive care unit (ICU), operating room, and cardiac catheterization laboratory; OHCA due to accidental serious hypothermia; and cardiac arrest with signs of potential recovery under the condition that protocols, staff, and equipment are available for initiating extracorporeal membrane oxygenation (ECMO) [1]. The International Liaison Committee on Resuscitation (ILCOR) has not reported cases of ECPR performed in children with OHCA. Therefore, no clear guidelines have been established [2]. In this article, we report a case of OHCA caused by fulminant myocarditis in a child. In this case, ECPR was performed by inserting catheters through the femoral artery and vein in the emergency outpatient unit, and a favorable outcome was achieved. We also summarize the clinical characteristics based on an analysis of the clinical course.

## Case presentation

The patient was a 10-year-old, innately healthy girl. She had developed symptoms of a common cold three days earlier. In the early morning on the day of admission, her mother observed that she had convulsions and called for an ambulance. She presumed that the girl had suffered cardiac arrest and immediately performed chest compression. Electrocardiography performed by emergency responders showed pulseless electrical activity (PEA). Since VT occurred during transportation, defibrillation was performed once. On arrival at the hospital, the waveform on the electrocardiographic monitor indicated VT. An emergency physician, pediatrician, and nurse attended to the patient. The emergency physician intubated the patient through the mouth, and the pediatrician secured a peripheral venous line. The emergency responders continued to perform cardiac massage for 37 min. Amiodarone and magnesium sulfate were administered intravenously, and defibrillation was performed. As the VT persisted, the emergency physician additionally called for a critical care physician, another nurse, and a clinical engineer to perform ECPR in the emergency outpatient unit. The critical care physician made an approximately 4 cm skin incision over the right inguinal region. The right femoral artery and vein were exposed, and 4-Fr sheaths were inserted. While the tip of the guidewire was confirmed using transthoracic echocardiography, an 18-Fr venous catheter was placed in the inferior vena cava. Next, the tip of a 13.5-Fr arterial cannula was placed near the junction between the abdominal aorta and the common iliac artery. At 67 min after the mother had found the girl in a state of cardiac arrest, extracorporeal circulation with venous artery extracorporeal membrane oxygenation (VA-ECMO) was initiated. ECMO was initially set at a rotation rate of 2330 bpm and a flow rate of 2.3 L/min. 

**Figure 1:**
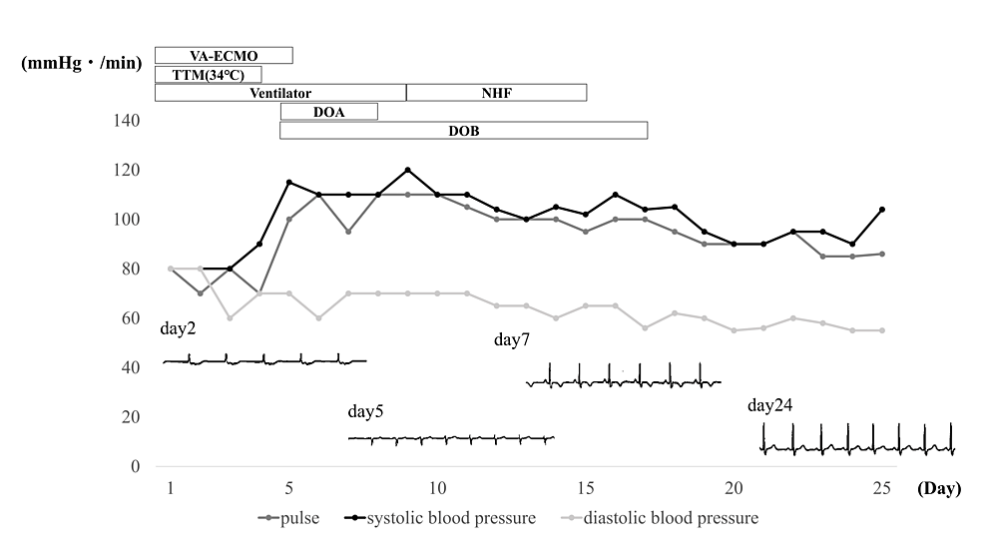
Progress after hospitalization. After ECMO initiation, the pulse pressure was not detected, and electrocardiography showed an atrioventricular junctional rhythm. Various electrocardiographic changes, such as premature atrial and ventricular contractions, were also observed. On day 3 of hospitalization, the pulse pressure and self-monitored pressure increased. On day 5 of hospitalization, the girl was weaned off VA-ECMO. On day 7 of hospitalization, a P wave was detected on an electrocardiogram. On day 9 of hospitalization, the patient was extubated and discharged from the intensive care unit. On day 24 of hospitalization, normal sinus rhythm was detected on an electrocardiogram. On day 25 of hospitalization, the patient was discharged. VA-ECMO, veno-arterial extracorporeal membrane oxygenation; TTM, targeted temperature management; NHF, nasal high flow; DOA, dopamine; DOB, dobutamine

Laboratory investigations of blood samples collected after the initiation of VA-ECMO showed an increase in creatine kinase (CK) level to 678 U/L, CK-Mb isoenzyme level to 117 U/L, and troponin I level to 16.94 ng/mL (Table 1). After the initiation of ECMO, a third defibrillation was performed (biphasic defibrillator 200 J), and atrioventricular junctional rhythm was achieved. Echocardiography revealed an ejection fraction of 10% and diffuse hypokinesia of the left ventricular wall. Whole-body CT showed no abnormalities. Fulminant myocarditis was diagnosed based on the preceding symptoms of the common cold, rapid circulatory failure, echocardiographic findings, electrocardiographic changes, and hematologic test results showing elevated levels of myocardial component proteins. After admission to the ICU, no pulse pressure was detected, and several electrocardiographic changes were observed. Targeted temperature therapy (TTM) at 34°C was administered for 24 h. Although hemorrhage was noted at the insertion sites of the VA-ECMO catheters, it was treated with compression and blood transfusion. No ischemia due to catheterization was observed in the lower limbs. On day 3 of hospitalization, her pulse pressure recovered. On day 5 of hospitalization, she was weaned off VA-ECMO after an increase in blood pressure and recovery of cardiac function were confirmed. On day 7 of hospitalization, a P wave was detected on the electrocardiogram. The patient was extubated on day 9 of hospitalization. On day 25 of hospitalization, the patient was discharged without neurological sequelae (Figure 1).

**Table 1:** Laboratory data. CRP, C-reactive protein; TP, total protein; Alb, albumin; UN, urea nitrogen; Cr, creatinine; AST, aspartate aminotransferase; ALT, alanine aminotransferase; LD, lactate dehydrogenase; ALP, alkaline phosphatase; γ-GT, gamma-glutamyltransferase; CK, creatine kinase; CK-MB, creatine kinase-MB; WBC, white blood cell; Hb, hemoglobin; Plt, platelet; PT, prothrombin time; APTT, activated partial thromboplastin time; FDP, fibrinogen degradation product; BE, base excess; Lac, lactate; AG, anion gap

Biochemical findings			Blood test		
CRP	0.1	mg/dL	WBC	8.0	10＾3/μL
Na	157	mmol/L	RBC	2.93	10＾3/μL
K	2.8	mmol/L	Hb	8.7	g/dL
Cl	105	mmol/L	PLT	82	10＾3/μL
T-P	3.3	g/dL	PT	21.7	seconds
Alb	1.9	g/dL	APTT	178.0	seconds
UN	19	mg/dL	D-dimer	430.8	μg/dL
Cr	0.80	mg/dL	Arterial blood gas		
AST	203	U/L	pH	7.491	
ALT	97	U/L	pCO_2_	29.5	mmHg
LDH	419	U/L	PO_2_	555	mmHg
γ-GTP	78	U/L	HCO_3_^-^	22.3	mmol/L
CK	678	U/L	BE	-0.2	mmol/L
CK-MB	117	U/L	Lactate	16	mmol/L
Troponin I	16.940	ng/mL	AG	22.3	mmol/L

## Discussion

The data based on which the recommendations for pediatric ECPR were published by the JRC and ILCOR were primarily collected from patients with a history of heart disease. ECPR is indicated for children with in-hospital cardiac arrest (IHCA) because many reports have shown that neurological outcomes are better with ECPR than with conventional cardiopulmonary resuscitation, and the survival rate is higher when ECPR is performed at a cardiac catheterization laboratory [3-4]. However, ECPR has been rarely performed in children with OHCA, and the evidence for the use of EPCR in OHCA is inadequate. Thus, ECPR is not recommended for children with OHCA, and the indications for its use in OHCA are unclear. Pediatric extracorporeal cardiopulmonary resuscitation (ECPR) ELSO Guidelines also state that the evidence is insufficient and that the provision of ECPR for OHCA should be done under a clear protocol [5]. Hence, the accumulation of data from each case of pediatric OHCA is important for establishing evidence.

The evidence for the use of ECPR in OHCA is inadequate. Thus, ECPR indications for pediatric OHCA have not been determined. In adults with OHCA, ECPR should be considered to improve neurological outcomes if certain criteria are met. According to the Study on Advanced Cardiac Life Support for Ventricular Fibrillation with Extracorporeal Circulation in Japan (SAVE-J), which was conducted among adults with OHCA who underwent ECPR in Japan, the criteria for initiating ECPR include (1) age 20-75 years, (2) initial electrocardiography showing ventricular fibrillation or pulseless VT, and (3) no response to standard advanced cardiac life support (ACLS). The criteria for not using ECPR were as follows: (1) the time to arrival at the emergency department (ED) was 45 min or longer after the episode of OHCA; (2) the patient responded to standard ACLS for 15 min after arrival at the ED; (3) the core temperature was less than 30°C on admission; (4) the levels of activities of daily living were poor before the onset of cardiac arrest; and (5) consent from the patient’s family was unavailable [6]. Due to the recent increase in reports of successful rescue in children with fulminant myocarditis, it is recommended that children be transported to institutions where ECPR can be initiated early [7]. Thus, in children with OHCA, initiation of ECPR should be considered when cardiac arrest is assumed to have been caused by heart diseases, such as fulminant myocarditis, or when the indications and contraindications for initiating ECPR in adults are met.

The ECPR issues for pediatric OHCA include cannulation. When ECPR is initiated in children, the selection of the cannula size and cannulation methods requires careful consideration due to the small diameters of the blood vessels. The extracorporeal life support organization (ELSO) recommends percutaneous placement of cannulae when they are inserted to perform ECMO in children aged ≥ 2 years [8]. Usually, when the percutaneous insertion is impossible, a cut-down procedure is performed. According to a study conducted by Burke et al., the time taken by experienced surgeons to initiate ECMO is 25 min for adults and 33 min for children [8]. Their study demonstrated that catheterization in children required a longer time than in adults. When the child’s body moves as the cardiac massage is performed, percutaneous catheterization of the small blood vessels is difficult. Moreover, performing an incision for catheterization requires approximately 20 min [8]. Since time is spent in obtaining the patient’s background information and deliberating the indication for ECPR in the initial treatment of OHCA, catheterization should be performed more rapidly. Thus, catheterization using a cut-down procedure should be considered in the early stages of ECPR in children.

In addition to the insertion methods, it is necessary to keep catheters in stock for children. In general, the selection of a short venous drainage catheter with a large diameter is recommended to achieve an appropriate flow rate in VA-ECMO. This is because reduced pressure loss improves venous drainage [9]. We used a Capiox percutaneous catheter kit (13.5 Fr) (CX-EB13ALX, Terumo, Tokyo, Japan) for the arterial line and a Capiox percutaneous catheter kit (18 Fr) (CX-EB18VLX, Terumo, Tokyo, Japan) for the venous line. These are catheters with the smallest diameter used for access from the thigh. For a 10-year-old Japanese girl with standard body size, it was possible to maintain the ECMO flow rate with this cannula size. However, information on cannula size for children under the age of 10 is unknown, and further case accumulation is required.

## Conclusions

There are no clear guidelines for the use of ECPR in children with OHCA. However, some children can be saved if ECPR is appropriately performed. Therefore, it is necessary to publish more cases of successful rescues. Based on our experience, the important factors for successful ECPR are: that the cause is cardiogenic, using the cut-down procedure as the first choice, leading to a rapid catheterization, and keeping stock of catheters of appropriate size, that should be selected.
